# Management of prepregnancy, pregnancy, and postpartum obesity from the FIGO Pregnancy and Non‐Communicable Diseases Committee: A FIGO (International Federation of Gynecology and Obstetrics) guideline

**DOI:** 10.1002/ijgo.13334

**Published:** 2020-09-07

**Authors:** Fionnuala M. McAuliffe, Sarah Louise Killeen, Chandni Maria Jacob, Mark A. Hanson, Eran Hadar, H. David McIntyre, Anil Kapur, Anne B. Kihara, Ronald C. Ma, Hema Divakar, Moshe Hod

**Affiliations:** ^1^ UCD Perinatal Research Centre School of Medicine National Maternity Hospital University College Dublin Dublin Ireland; ^2^ Institute of Developmental Sciences Faculty of Medicine University of Southampton Southampton UK; ^3^ NIHR Southampton Biomedical Research Centre University Hospital Southampton Southampton UK; ^4^ Maternal‐Fetal Medicine Unit Rabin Medical Center Petach‐Tikva Israel; ^5^ Sackler Faculty of Medicine Tel Aviv University Tel‐Aviv Israel; ^6^ Mater Research The University of Queensland South Brisbane Qld Australia; ^7^ World Diabetes Foundation Bagsværd Denmark; ^8^ African Federation of Obstetricians and Gynaecologists Khartoum Sudan; ^9^ Department of Obstetrics and Gynecology School of Medicine University of Nairobi Nairobi Kenya; ^10^ Department of Medicine and Therapeutics The Chinese University of Hong Kong Hong Kong SAR China; ^11^ Hong Kong Institute of Diabetes and Obesity The Chinese University of Hong Kong Hong Kong SAR China; ^12^ Divakars Speciality Hospital Bengaluru India; ^13^ Mor Comprehensive Women’s Health Care Center Tel Aviv Israel; ^14^ FIGO Pregnancy and Non‐Communicable Diseases Committee International Federation of Gynecology and Obstetrics London UK

## 1 Executive Summary

Obstetricians and gynecologists are well positioned to influence population health through maternity and women’s health services. Obesity is common in women of reproductive age and the prevalence is rising in both low‐/middle‐income and high‐income countries^1^. Obesity affects requirements for assessment, monitoring, and intervention and can impact maternal and child outcomes. Obstetricians and gynecologists require guidance on the care of women of reproductive age with obesity at all time points related to pregnancy, including how to address modifiable risk factors such as diet and physical activity. Many guidelines have been developed to date, although they vary in scope, methodology, and individual recommendations.

FIGO’s Committee Guideline for the Management of Prepregnancy, Pregnancy, and Postpartum Obesity (Table 1) reviews good clinical practice recommendations (Table 2, 3, 4) from previously published international documents. It serves as a practical resource to support obstetricians and gynecologists in the management of women with obesity. We emphasize the role of clinicians in preventing and managing obesity for women before, during, and after pregnancy, harnessing the increased contact with healthcare professionals during this period. FIGO expects that the recommendations in this guideline will inform the development of evidence‐based, country‐specific guidance on the management of obesity in member organizations, that are in line with local needs, practices, policies, and available resources.

**Table 1:** FIGO Committee guideline for the management of prepregnancy, pregnancy, and postpartum obesity

Time point A: Prepregnancy	
A.1	All women should have their weight and height measured and their body mass index (BMI, calculated as weight in kilograms divided by height in meters squared) calculated. Consider ethnic differences.
A.2	All women with a BMI of ≥30 should be advised of the effect of obesity on fertility, the immediate risks of obesity during pregnancy and childbirth, and the subsequent long‐term health effect of obesity including the higher risk of noncommunicable diseases for them and their children.
A.3	All women with obesity should be encouraged to lose weight through diet and adopting a healthy lifestyle including moderate physical activity. If indicated and available, other weight management interventions might be considered, including bariatric surgery.
A.4	All women with obesity should be advised to take at least 0.4 mg (400 μg) and consider up to 5 mg folic acid supplementation daily for at least 1–3 months before conception.
**Time point B: Pregnancy**	
B.1	All women should have their weight and height measured and their BMI calculated at the first antenatal visit. Consider ethnic differences. Advise on appropriate gestational weight gain.
B.2	All women should receive information on diet and lifestyle appropriate to their gestation including nutrient supplements, weight management, and regular physical activity.
B.3	All women with obesity should be advised of the risks of obesity and excess gestational weight gain on pregnancy, childbirth, and long‐term health including risk of noncommunicable diseases for them and their children.
B.4	All antenatal healthcare facilities should have well‐defined multidisciplinary pathways for the clinical management of pregnant women with obesity including the identification and treatment of pregnancy‐related complications.
**Time point C: Postpartum**	
C.1	All women with prepregnancy obesity should receive support on breastfeeding initiation and maintenance.
C.2	All women with obesity and pregnancy complications should receive appropriate postnatal follow‐up in line with local resources, care pathways, and in response to the individual health requirements of each woman and her children.
C.3	All women with obesity should be encouraged to lose weight post partum with emphasis on healthy diet, breastfeeding if possible, and regular moderate physical activity. They should be advised of the importance of long‐term follow‐up as they and their children are at increased risk for noncommunicable diseases.
C.4	Maternal obesity should be considered when making the decision regarding the most appropriate form of postnatal contraception.

**Table 2:** Good clinical practice recommendations for prepregnancy obesity (timepoint A)

Recommendation		Strength
A.1.1	Primary care services should support women of childbearing age with weight management before pregnancy and body mass index (BMI, calculated as weight in kilograms divided by the square of height in meters) should be measured. Advice on weight and lifestyle should be given during periodic health examinations, preconception counselling, contraceptive consultations, or other gynecologic care prior to pregnancy.	Conditional 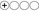
A.2.1	Women of childbearing age with obesity should receive information and advice about the effect of obesity on fertility and the risks of obesity during pregnancy and childbirth.	Conditional 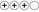
A.2.2	Assessment for sleep apnea and other conditions that could affect health during pregnancy, including those of the cardiac, pulmonary, renal, endocrine, and skin systems is warranted in the preconception period.	Conditional 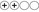
A.3.1	Weight management strategies prior to pregnancy could include dietary, exercise, medical, and surgical approaches. Diet and exercise are the cornerstone of weight management in preconception and pregnancy.	Conditional 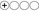
A.4.1	Women with a BMI ≥30 wishing to become pregnant should be advised to take a folic acid supplement daily, starting at least 1–3 months before conception and continuing during the first trimester of pregnancy. The dose should be at least 0.4 mg (400 μg) and consideration should be given to a higher dose (5 mg) as obesity is a risk factor for neural tube defects.	Strong 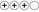

**Table 3:** Good clinical practice recommendations for pregnancy obesity (timepoint B)

Recommendation		Strength
B.1.1	All pregnant women should have their height and weight measured at their first antenatal visit. This can be used to calculate body mass index (BMI, calculated as weight in kilograms divided by the square of height in meters). Data should be recorded in the medical records.	Conditional 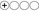
B.1.2	Approaches to monitor and manage gestational weight gain should be integrated into routine antenatal care practices.	Strong 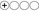
B.1.3	Pregnant women with a BMI ≥30 should be advised to avoid high gestational weight gain. Weight gain should be limited to 5–9 kg.	Strong 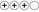
B.2.1	The mainstay of weight management during pregnancy is diet and exercise. Health professionals should provide general nutrition information and advice on a healthy diet to manage weight during pregnancy. Where resources permit, individual plans for diet and exercise for weight management should be put in place.	Conditional 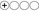
B.2.2	Moderate intensity and appropriate exercise should be encouraged during pregnancy.	Strong 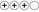
B.2.3	Women with obesity should continue to take folic acid during at least the first trimester.	Strong 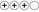
B.2.4	Women with previous bariatric surgery require closer screening and monitoring of their nutritional status and fetal growth throughout pregnancy. They should be referred to a dietitian for advice about their nutritional needs and, where possible, have consultant‐led care.	Conditional 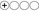
B.3.1	All pregnant women with obesity in early pregnancy should be provided with accurate and accessible information about the risks associated with obesity and how they may be minimized.	Conditional 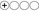
B.3.2	Women should be informed that some screening processes for chromosomal anomalies are less effective in obesity.	Strong 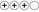
B.3.3	All pregnant women should be advised individually on mode of delivery, considering the risk of emergency cesarean delivery.	Conditional 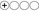
B.4.1	Where possible, healthcare facilities should have clearly defined pathways for the management of pregnant women with obesity. The adequacy of resources and equipment available should be considered when making decisions around care, especially for women with a BMI ≥40.	Conditional 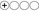
B.4.2	Women with obesity with multiple gestations require increased surveillance and may benefit from consultation with a maternal–fetal medicine consultant.	Strong 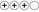
B.4.3	All pregnant women with a BMI ≥30 should be screened for gestational diabetes in early pregnancy.	Strong 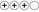
B.4.4	Where available, an appropriately sized blood pressure cuff should be used for measurements. The cuff size used at the earliest time point should be documented in the medical records.	Conditional 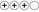
B.4.5	To help prevent pre‐eclampsia, prophylactic aspirin from early pregnancy can be recommended to women with obesity who have other moderate to high risk factors.	Strong 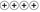
B.4.6	Clinicians should be aware that women with a BMI ≥30, before pregnancy or in early pregnancy, have a pre‐existing risk factor for developing venous thromboembolism during pregnancy. Risk of antenatal and postnatal venous thromboembolism should be assessed.	Strong 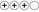
B.4.7	If available, women with a BMI ≥35 should be referred for serial assessment of fetal size using ultrasound as they are more likely to have inaccurate symphysis–fundal height measurements.	Conditional 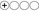
B.4.8	Due to the elevated risk of stillbirth associated with obesity, greater fetal surveillance is recommended in the third trimester in the case of reduced fetal movements.	Conditional 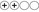
B.4.9	Women with a BMI ≥30 are at increased risk of mental health problems, including anxiety and depression. Healthcare professionals should offer psychological support, screen for anxiety and depression, and refer for further support where appropriate and available.	Strong 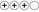
B.4.10	Induction of labor is recommended at 41^+0^ weeks of gestation for women with a BMI ≥35 owing to their increased risk of intrauterine death.	Strong 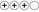
B.4.11	Women with a BMI ≥40 should be referred to an anesthetist for assessment in the antenatal period.	Conditional 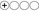
B.4.12	Electronic fetal monitoring is recommended for women in active labor with a BMI ≥35. Intrauterine pressure catheters and fetal scalp electrodes may help.	Conditional 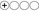
B.4.13	In the case of vaginal delivery for women with a BMI ≥40, early placement of an epidural catheter is advisable in the case of an emergency cesarean delivery.	Conditional 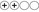
B.4.14	Establish venous access in early labor for women with a BMI ≥40 and consider a second cannula.	Conditional 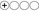
B.4.15	Women with a BMI ≥30 having a cesarean delivery are at increased risk of wound infection and should receive prophylactic antibiotics at the time of surgery. Women with obesity may benefit from higher doses.	Strong 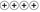
B.4.16	Active management of the third stage should be recommended to reduce the risk of postpartum hemorrhage.	Strong 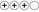
B.4.17	Postoperative pharmacologic thromboprophylaxis should be prescribed based on maternal weight.	Conditional 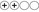
B.4.18	Mechanical thromboprophylaxis is recommended before and after cesarean delivery. Where available, women with a BMI ≥35 should be given graduated compression stockings, or other interventions such as sequential compression devices, after cesarean delivery until mobilization, which should be encouraged early.	Conditional 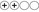

**Table 4:** Good clinical practice recommendations for postpartum obesity (timepoint C)

Recommendation		Strength
C.1.1	Obesity is associated with low breastfeeding initiation and maintenance. Women with obesity in early pregnancy should receive specialist advice on the benefits of breastfeeding and appropriate antenatal and postnatal support for breastfeeding initiation and maintenance.	Conditional 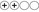
C.2.1	Women with obesity who have been diagnosed with gestational diabetes and other pregnancy complications should have appropriate postnatal follow‐up.	Conditional 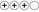
C.2.2	Due to the increased risk associated with obesity, where available, women with obesity should be screened for postpartum mental health disorders such as depression and anxiety.	Strong 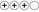
C.3.1	Women should be informed that weight loss between pregnancies reduces the risk of stillbirth, hypertensive complications, and fetal macrosomia in subsequent pregnancies. Weight loss increases the chances of successful vaginal birth after cesarean delivery.	Strong 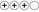
C.3.2	Women with obesity should be offered further dietary and physical activity advice to support postpartum weight management.	Conditional 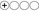
C.4.1	Women with obesity should be counselled on the most appropriate form of postnatal contraception based on BMI.	Conditional 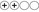

## 2 Introduction

### 2.1 Significance of obesity in women before, during, and after pregnancy

Obesity has become the most common medical condition in women of reproductive age and the rise in prevalence of obesity is seen in both high‐income countries and low‐/middle‐income countries (LMICs)^1^. It is predicted that by 2025 more than 21% of women in the world will have obesity^2^. In the USA, 2011–2012 NHANES (National Health and Nutrition Examination Survey) data indicate that the prevalence of obesity in women aged 20–39 years is at least 31.8% and is even higher in women of low incomes at 61%^3^. The prevalence of maternal obesity varies in different African nations, ranging from 17.9% in the first trimester and up to 6.5%–50.7% in the third trimester^4^. Routine surveillance of weight gain during pregnancy is not conducted in many countries. However, body mass index (BMI) among women in the reproductive age group is used often as an indicator of maternal obesity and its likely effect on pregnancy outcomes and subsequent health of the woman and her child^1^.

Obesity increases the risk of noncommunicable diseases (NCDs), such as type 2 diabetes and cardiovascular disease, which contribute to over 70% of global deaths annually^5^, ^6^. This is especially important in LMICs where 86% of premature NCD deaths occur^7^. Increasing evidence from the developmental origins of health and disease paradigm suggests that obesity during pregnancy not only increases the mother’s risk of later NCDs but can also transfer the risk to the offspring through epigenetic mechanisms, alterations in gut microbiome, and sociocultural factors^8^. In addition, excessive gestational weight gain during pregnancy can result in further elevated maternal BMI in subsequent pregnancies if weight loss is not achieved in the postpartum period, particularly in the first 6–12 months^9^, ^10^.

Comorbidities such as gestational diabetes mellitus (GDM) are more common in pregnant women with obesity, and this not only increases the risk of subsequent type 2 diabetes mellitus for the mother but also leads to increased fetal growth, large‐for‐gestational‐age babies, and metabolic compromise in the offspring^11^, ^12^, ^13^, ^14^. These outcomes in offspring are further associated with long‐term consequences such as childhood obesity and type 2 diabetes mellitus in later life^15^. Other long‐term consequences include poor cognitive performance in the child and neurodevelopmental disorders including cerebral palsy^8^. Pregnant women with obesity are also more likely to deliver by cesarean and have difficulties initiating breastfeeding and a reduction in duration of breastfeeding^1^, ^16^. Obesity in pregnant mothers is also associated with immediate adverse outcomes such as stillbirth with risk increasing with higher maternal BMI^17^. Finally, maternal obesity places women at a higher risk of infertility^18^. Women with obesity are more likely to have issues with ovulation or endometrial function, and weight loss in women with obesity is associated with improved fertility^19^, ^20^.

### 2.2 Role of healthcare professionals in obesity management

The causes of maternal obesity are multifaceted, including societal, environmental, and other factors, calling for a multisystem, life course approach to obesity prevention and management. However, obstetricians and gynecologists are uniquely positioned to influence obesity risk and prevalence through lifestyle and other interventions with women of reproductive age, before, during, and after pregnancy. The preconception and postpartum periods are opportunities for intensive nutrition and weight optimization, while during pregnancy, the focus should be on appropriate gestational weight gain while meeting nutritional requirements. Postpartum weight retention can significantly alter the weight gain trajectory of a woman during childbearing years, especially in the case of multiple pregnancies. The preconception and postpartum periods are thus key time points for weight management strategies to delay or prevent the development or progression of obesity in women, promote the health of women in pregnancy, and reduce the risks passed to future generations^9^.

Women with obesity during pregnancy require specialist care and recommendations for this are outlined in this article. Maternity costs are higher for women with obesity due to the increased and specialized requirements associated with care and increased use of healthcare services among women with obesity^21^. Such issues highlight the additional importance of nutrition and weight management in the preconception and postpartum periods and to increase awareness about optimal gestational weight gain among clinicians and the general population.

While healthcare models and care pathways for women before, during, and after pregnancy vary internationally, obstetricians/gynecologists and midwives are well positioned to influence population health through maternity and women’s health services. However, several barriers exist in delivering these preventive services during routine clinical practice. Often, time constraints during appointments can hamper effective discussions related to nutrition and weight management during antenatal visits^22^, ^23^. A recent review of midwives’ and obstetricians/gynecologists’ knowledge of gestational weight gain guidelines showed that, overall, healthcare professionals demonstrated inadequate knowledge of these guidelines^24^. Self‐reported knowledge was higher than directly assessed knowledge and a pooled analysis was difficult owing to differences in guidelines between countries. In addition, cultural influences may determine the healthcare professional’s subjective perception of body image and weight and, in the absence of anthropometric measurement and classification, this could result in underestimation and assessment of weight‐related risks. Resources such as the FIGO Nutrition Checklist and guidance may assist obstetricians and gynecologists by increasing professional knowledge and time management when caring for women^21^, ^22^, ^25^, ^26^, ^27^.

### 2.3 FIGO guidance for the management of prepregnancy, pregnancy, and postpartum obesity

Considering the increasing global rates of obesity during pregnancy and their long‐term effects for the health of the mother and the next generation, it is essential to address the issue of gestational weight gain. The authors reviewed existing clinical guidelines for the management of women with obesity before, during, and after pregnancy and identify key recommendations for global clinical practice. The Pregnancy Obesity and Nutrition Initiative (PONI) developed by FIGO’s Pregnancy and Non‐Communicable Diseases (PNCD) Committee emphasizes that management of obesity in pregnancy should be considered in the context of a life course approach, linking with preconception and postpartum and interconception services to prevent excess weight gain before and during pregnancy^5^. PONI also aims to support healthcare professionals such as obstetricians/gynecologists, midwives, nurses, dietitians, and endocrinologists to develop collaborative action to prevent and reduce the burden of maternal obesity. This guidance also outlines potential actions to address the barrier of ineffective communication of risks related to maternal obesity. To date, over 30 clinical practice guidelines have been published internationally, which focus specifically on or incorporate some guidance on the management of women with obesity during pregnancy^28^. These guidelines vary in scope, evidence quality, and international relevance. Therefore, this guidance for the management of prepregnancy, pregnancy, and postpartum obesity consolidates recommendations from multiple practice guidelines and suggests a pragmatic approach for the management of obesity in women before, during, and after pregnancy.

### 2.4 Target audience

This guidance is directed at healthcare providers working with women with obesity, before, during, and after pregnancy. Obesity management may require and benefit from the involvement of a variety of professionals such as general practitioners/family physicians, midwives, nurses, community health workers, dietitians, nutritionists, physiotherapists, and others^29^. A multidisciplinary approach to obesity management before, during, and after pregnancy is therefore recommended. The guidance outlined here is relevant to individual practitioners providing primary care, gynecological care, and support to women during pregnancy and outside of pregnancy, and their respective professional organizations. This guidance is also relevant to healthcare delivery organizations and providers as it may guide and give insight into resource requirements for this group. This includes governments, legislators, healthcare or insurance organizations, and development agencies. The article focuses on three key time points, related to pregnancy: prepregnancy (time point A), pregnancy (time point B), and the postpartum period (time point C).

For the purpose of this review, obesity is defined in terms of BMI (calculated as weight in kilograms divided by the square of height in meters). According to the World Health Organization (WHO), a BMI score greater than 30 is considered to indicate obesity^30^. Obesity is further divided into three classes of increasing severity: obesity class I 30–34.9; class II 35–39.9; and class III greater than 40. The potential influence of ethnicity on BMI, body fat percentage, and associated risk factors for chronic disease should be considered and the general cut‐offs outlined above may not be the most suitable for all populations, including some Asian populations^30^. The WHO recommends that each country make a decision on risk and desired BMI cut‐offs based on the characteristics of their specific population and have identified a number of points along the BMI continuum that may indicate elevated risk in Asian populations. In some cases, a BMI of 23 or 27.5 may be a relevant marker of risk, in comparison to BMI of 25 and 30^30^, ^31^. FIGO member states using this resource should consider the specific BMI cut‐offs that are appropriate for their population to identify women with obesity. The recommendations in this guideline will likely be relevant to the management of women with obesity, regardless of which BMI cut‐off is used to define this risk.

While this article focuses on the management of women with obesity, several of the risks associated with obesity likely start to increase when BMI enters the overweight category (usually defined as BMI 25–29)^32^, ^33^. Therefore, the advice may also be useful for women with a BMI in the overweight category who are at increased risk of adverse health outcomes and who may benefit from the diet, physical activity, and other interventions outlined throughout^33^.

### 2.5 Identification of recommendations and evidence grading

Published clinical practice guidelines that focus specifically on obesity in pregnancy and include evidence grading were considered for this review. Sources include the recent systematic review on published clinical practice guidelines on obesity in pregnancy^28^, supplemented with an independent review of grey literature, including the Geneva Foundation for Medical Education and Research^34^. To be included in the current review, a guideline needed to originate from a FIGO member organization, focus primarily on the management of pregnancy obesity rather than associated comorbidities such as gestational diabetes mellitus, and include some form of evidence grading to qualify recommendations. The recommendations from included guidelines were extracted, along with their evidence grading. Further details of the methods involved in this review are included in supporting information S1.

There was variance in the evidence grading systems used by the clinical practice guidelines, and evidence gradings were translated to the GRADE (Grading of Recommendations Assessment, Development and Evaluation) framework for consistency, based on the information in each of the clinical practice guidelines (supporting information S2)^35^. The interpretation of this can be seen in Tables 5 and 6. The sources of recommendations are referenced throughout this article and should be consulted for more details on the individual studies involved in the generation of the level of evidence qualifier. The clinical practice guidelines were reviewed in order of publication. Where conflicting guidance or evidence grading was provided, the guidance from the most recently published clinical practice guideline is outlined. This approach was taken as it best reflects the most up‐to‐date evidence. Each recommendation or set of related recommendations is followed by a summary of the rationale and evidence and remarks or caveats for operationalizing it in diverse settings, where relevant. For full details of the list of recommendations and corresponding evidence in each of the guidance documents, the specific reference document should be reviewed. The final advice in this document was agreed upon through independent review by members of the FIGO PNCD Committee and consolidation within the committee.

**Table 5:** Interpretation of strong and conditional (weak) recommendations according to GRADE.^a^, ^b^

Implications	1 = Strong recommendation phrased as “we recommend”	2 = Conditional (weak) recommendation phrased as “we suggest”
For patients	Nearly all patients in this situation would accept the recommended course of action. Formal decision aids are not needed to help patients make decisions consistent with their values and preferences	Most patients in this situation would accept the suggested course of action
For clinicians	According to the guidelines, performance of the recommended action could be used as a quality criterion or performance indicator	Decision aids may help patients make a management decision consistent with their values and preferences
For policy makers	The recommendation can be adapted as policy in most situations	Stakeholders need to discuss the suggestion

^a^ Adapted with permission of the American Thoracic Society. © 2020 American Thoracic Society. Schunemann HJ, Jaeschke R, Cook DJ, et al. An official ATS statement: grading the quality of evidence and strength of recommendations in ATS guidelines and recommendations. Am J Respir Crit Care Med 2006;174:605–614. The American Journal of Respiratory and Critical Care Medicine is an official journal of the American Thoracic Society. Readers are encouraged to read the entire article for the correct context at www.atsjournals.org/journal/ajrccm. The authors, editors, and The American Thoracic Society are not responsible for errors or omissions in adaptations.

^b^ Both caregivers and care recipients need to be involved in the decision‐making process before adopting recommendations.

**Table 6:** Interpretation of quality of evidence levels according to GRADE.^a^

Level of evidence	Definition
High 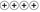	We are very confident that the true effect corresponds to that of the estimated effect
Moderate 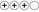	We are moderately confident in the estimated effect. The true effect is generally close to the estimated effect, but it may be slightly different
Low 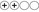	Our confidence in the estimated effect is limited. The true effect could be substantially different from the estimated effect
Very low 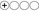	We have very little confidence in the estimated effect. The true effect is likely to be substantially different from the estimated effect

^a^ Adapted with permission from Balshem et al. GRADE guidelines: 3. Rating the quality of evidence. J Clin Epidemiol 2011;64:401–6. © 2011 Elsevier.

## 3 FIGO Guidance for Prepregnancy Obesity

*Recommendation A.1. All women should have their weight and height measured and their body mass index calculated at each contact.*

**Table:** 

A.1.1. Primary care services should support women of childbearing age with weight management before pregnancy and BMI should be measured. Advice on weight and lifestyle should be given during periodic health examinations, preconception counselling, contraceptive consultations, or other gynecologic care prior to pregnancy. **Conditional** 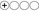

The preconception and interpregnancy periods are good opportunities for safe weight management interventions. As many women of childbearing age may not attend preconception health services, other health presentations such as regular health examinations, routine gynecological appointments, and other primary care services should be utilized to check and monitor weight^36^. Weight and height should be measured and BMI calculated for all women of childbearing age to encourage them, if needed, to optimize their weight before pregnancy^37^, ^38^In addition, women with obesity may experience higher rates of contraceptive failure and thus may not present to healthcare services for pregnancy planning^38^, ^39^. Consideration should be given to weight monitoring in routine health checks and clinical presentations so that the individual weight trajectory of a woman can be considered.

*Recommendation A.2. All women with a BMI of ≥30 should be advised of the effect of obesity on fertility, the immediate risks of obesity during pregnancy and childbirth, and the subsequent long‐term health effect of obesity including the higher risk of NCDs for them and their children.*

**Table:** 

A.2.1. Women of childbearing age with obesity should receive information and advice about the effect of obesity on fertility and the risks of obesity during pregnancy and childbirth. **Conditional** 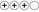

Women with obesity may be unaware of the extent of adverse health outcomes of pregnancy that are related to obesity and may hold misconceptions about the relationship between diet, weight, and health in pregnancy^40^, ^41^, ^42^. Advice should be given to women with obesity of the effect on their fertility and the risks of obesity during pregnancy and childbirth, which include neural tube defects, macrosomia, preterm delivery, stillbirth, shoulder dystocia, cesarean delivery, GDM, metabolic syndrome, sleep apnea, hypertension, and thromboembolic disorders^37^, ^43^, ^44^, ^45^.

**Table:** 

A.2.2. Assessment for sleep apnea and other conditions that could affect health during pregnancy, including those of the cardiac, pulmonary, renal, endocrine, and skin systems is warranted in the preconception period. **Conditional** 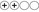

Obesity influences many body systems. The preconception period is an ideal time to assess and manage conditions that could influence the health of the mother and fetus during pregnancy^25^, ^43^. According to the Society of Obstetricians and Gynaecologists of Canada (SOGC), baseline preconception health screening in women with obesity, where available and indicated, could include renal, liver, and thyroid function, lipids, and diabetes screen. Additional considerations include cardiovascular health (e.g. blood pressure, echocardiogram) and pulmonary function tests (including sleep apnea where relevant). For further information see the FIGO position paper on preconception health^25^.

*Recommendation A.3. All women with obesity should be encouraged to lose weight through diet and adopting a healthy lifestyle including moderate physical activity. If indicated and available, other weight management interventions might be considered, including bariatric surgery.*

**Table:** 

A.3.1. Weight management strategies prior to pregnancy could include dietary, exercise, medical, and surgical approaches. Diet and exercise are the cornerstone of weight management in preconception and pregnancy. **Conditional** 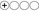

Women should be encouraged to enter pregnancy with a BMI <30, and ideally in the healthy range^36^. There is evidence that preconception weight loss, achieved through one or more nonsurgical or surgical interventions, has the potential to improve maternal health and reduce risk of pregnancy complications, even when the weight loss is small^43^, ^46^. Losing weight before pregnancy can have positive effects such as the reduced risk of obesity in children and improved fertility^8^, ^47^.

A realistic target is weight loss generally considered to be 5%–10% of original body weight over a period of 6 months^43^, ^48^, ^49^. Individualized counselling sessions that include dietary modifications for weight loss and optimizing nutritional status, combined with aerobic and strengthening exercises, should be considered as the first‐line therapy for the management of obesity before conception^46^, ^49^. Diet and lifestyle advice should be practical, implementable, and communicated clearly using plain and simple language^50^. Techniques such as goal setting, social support, and self‐monitoring may support the success of diet and lifestyle interventions for weight loss^51^.

Women with obesity are more likely to have nutritional deficiencies such as in vitamin D, iron, and vitamin B12, when compared to women with lower BMIs^37^. This may be due to reduced diet quality^52^. Dietary advice for women with obesity before pregnancy should focus on achieving weight loss or preventing further weight gain through nutrient‐dense foods, following local dietary practices and guidelines that are appropriate for age, medical history, and other characteristics of the woman e.g. dietary practices, allergies, intolerances, or other specific requirements. The specific foods to recommend will vary depending on national guidance, cultural practices, and other considerations^25^. Detailed information on diet before conception can be found in the FIGO recommendations on adolescent, preconception, and maternal nutrition^38^.

Due to insufficient safety data, medications and surgery for weight loss are not recommended around the time of conception, though should be considered as part of the management of obesity in women who are planning a pregnancy in the future, especially for those with comorbidities and greater severity obesity^33^, ^44^, ^53^. The degree of weight loss achievable with different interventions varies and practitioners and pregnant women should be aware of this in managing expectations for weight management^46^. Despite this, health benefits such as improved fertility are likely even with small amounts of weight loss and therefore all weight loss should be encouraged, even when achieving a healthy BMI before pregnancy is not possible^47^.

Finally, factors related to weight management, such as depression, should be considered and addressed where appropriate to support weight loss before conception^44^.

If preconception weight loss interventions include bariatric surgery, where possible, women should wait at least 12–18 months after treatment to conceive^54^. This is to allow for body weight stabilization and the identification and treatment of any nutritional deficiencies, especially in the case of malabsorptive surgery^44^, ^55^. Therefore, appropriate contraceptive advice should be given^55^. Women after bariatric surgery have been found to have any of a variety of vitamin and mineral deficiencies in pregnancy including vitamins A, C, D, B1, B6, B12, and K, iron, calcium, selenium, and phosphorous^56^. In fact, long‐term periodic screening for vitamin and mineral deficiencies is recommended for women after bariatric surgery along with prescribed multivitamin and mineral supplementation^57^. In cases of advanced maternal age, a shorter interval after surgery could be considered, including the effect of nutritional and other obesity‐related issues that are associated with a reduced time to pregnancy^54^. Due to the associated complexities of nutritional requirements in addition to the usual preconception needs, where possible women with a history of bariatric surgery should be referred to a dietitian before pregnancy^37^, ^49^

*Recommendation A.4. All women with obesity should be advised to take at least 0.4 mg (400 μg) and consider up to 5 mg folic acid supplementation daily for at least 1–3 months before conception.*

**Table:** 

A.4.1. Women with a BMI ≥30 wishing to become pregnant should be advised to take a folic acid supplement daily, starting at least 1–3 months before conception and continuing during the first trimester of pregnancy. The dose should be at least 0.4 mg (400 μg) and consideration should be given to higher dose (5 mg) as obesity is a risk factor for neural tube defects. **Strong** 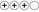

Periconceptional folic acid supplementation reduces the incidence of neural tube defects across resource settings^58^. While there are some differences within published guidelines, FIGO’s advice is that all women of childbearing age should consume at least 0.4 mg (400 μg) supplementary folic acid a day for at least 1 month before conception, continuing until at least the end of the first trimester of pregnancy, to reduce the risk of neutral tube defects^33^, ^44^, ^59^. Consideration should be given to the potential use of a higher dose (5 mg/d) for women with obesity, for example in those who may have other additional risk factors for neural tube defects, for example pregestational diabetes or family history, in line with local and national guidelines and resources^37^, ^60^, ^61^.

Depending on the specific diets of the population, women who could become pregnant or are planning a pregnancy may require additional vitamin and mineral supplements. A daily iodine supplement of 150 mg per day, for example, is recommended by the Royal Australian and New Zealand College of Obstetricians and Gynaecologists (RANZCOG) for women before conception^44^. Other guidelines suggest consideration of a daily vitamin D supplement^43^. Vitamin D supplementation may help ensure women with obesity are replete in Vitamin D; however, there is insufficient evidence to determine whether routine supplementation is required^37^. Dietary intakes, medical history, and other factors such as sun exposure should be considered.

## 4 FIGO Guidance for Pregnancy Obesity

*Recommendation B.1. All women should have their weight and height measured and their BMI calculated at the first anetanal visit. Consider ethnic differences. Advise on appropriate gestational weight gain.*

**Table:** 

B.1.1. All pregnant women should have their height and weight measured at their first antenatal visit. This can be used to calculate BMI. Data should be recorded in the medical records. **Conditional** 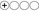

Weight and height should be measured and recorded at the first antenatal visit^37^. These measures should be used to calculate BMI. This helps give an indication of the pregnancy risks and management requirements. As outlined previously, prepregnancy or early‐pregnancy BMI of ≥30 is considered to indicate obesity, however consideration should be given to the health and other risks for women of Asian ethnicity with lower BMIs^30^. The frequency of subsequent weight checks should be conducted in line with local policy and practices and in consideration of the individual needs and circumstances of the woman.

**Table:** 

B.1.2. Approaches to monitor and manage gestational weight gain should be integrated into routine antenatal care practices. **Strong** 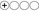

Recent evidence illustrates that gestational weight gain is an independent risk factor for adverse health outcomes and that it has a cumulative effect, whereby mothers with obesity who gain excessive weight during pregnancy have the greatest risk of pregnancy complications^43^, ^46^, ^62^, ^63^.

**Table:** 

B.1.3. Pregnant women with a BMI ≥30 should be advised to avoid high gestational weight gain. Weight gain should be limited to 5–9 kg. **Strong** 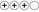

While the amount of gestational weight gain considered “normal” will vary regionally, consider advising pregnant women with obesity and a singleton pregnancy to limit gestational weight gain to approximately 5–9 kg to reduce the risk of adverse pregnancy outcomes^43^, ^45^, ^64^. This is based on the Institute of Medicine guidelines (IOM)^65^. Weight gain in excess of the IOM guidelines has been shown to increase risk of many pregnancy complications, including macrosomia, and cesarean delivery^66^. Some research suggests however, that the risk of pregnancy complications may be further reduced by limiting gestational weight gain to lower than 5–9 kg^67^. In addition, excessive gestational weight gain has been associated with adverse cardiometabolic outcomes in offpring^68^.

The IOM guidelines were developed based on the ethnic diversity in the USA in 1990 and 2009 and outline ranges of acceptable gestational weight gain according to weight definitions that are based on standard BMI cut‐offs, used by the WHO^65^, ^69^. In the absence of country‐specific recommendations based on individual population characteristics and trends, the IOM guidelines are used widely internationally, such as in Brazil, Malawi, Australia, and New Zealand^44^, ^70^, ^71^. The utility of the IOM guidelines for women of Indian and other Asian backgrounds has been questioned, mostly due to the variance in BMI cut‐offs used for this group^72^. Despite this, a recent systematic review found that gestational weight gain in excess of the IOM guidelines in Asian women was associated with adverse maternal and fetal outcomes^72^.

Some Asian countries have their own country‐specific guidelines, such as Japan, where national guidelines recommend lower gestational weight gain ranges than the IOM (≤5 kg)^73^. It is therefore prudent that in the absence of suitable country‐specific guidance, the IOM guideline of 5–9 kg gestational weight gain can be used for women with obesity. The classification of obesity should, however, be based on the BMI cut‐offs most appropriate to that country. This supports the correct application and interpretation of the guidelines and makes them suitable for widespread use.^73^, ^74^, ^75^

Recently, high‐quality large‐scale randomized controlled trials have reported that lifestyle interventions during pregnancy that include diet and exercise advice and behavior change support can reduce excessive gestational weight gain and the frequency of large‐for‐gestational‐age babies (LIMIT, UPBEAT, PEARS trials)^76^, ^77^, ^78^. Lifestyle interventions during pregnancy that include diet and physical activity have also been shown to reduce the risk of pregnancy‐induced hypertension, cesarean delivery, and respiratory distress in neonates^79^. Other interventions incorporating dietary counselling and low glycemic index dietary advice and supervised exercise have shown promise in preventing the transmission of risk factors for childhood obesity^77^, ^80^.

*Recommendation B.2. All women should receive information on diet and lifestyle appropriate to their gestation including nutrient supplements, weight management, and regular physical activity.*

**Table:** 

B.2.1. The mainstay of weight management during pregnancy is diet and exercise. Health professionals should provide general nutrition information and advice on a healthy diet to manage weight during pregnancy. Where resources permit, individual plans for diet and exercise for weight management should be put in place. **Conditional** 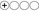

Diet and lifestyle interventions are clinically effective in reducing gestational weight gain and preventing pregnancy complications so should be offered to women with obesity. Women with obesity may have a higher prevalence of nutritional inadequacies, and intakes of certain micronutrients have been shown to correlate with BMI^81^. Dietary advice should therefore emphasize healthy dietary patterns that consist of nutrient‐dense foods. This may be characterized by higher intakes of fruit, vegetables, wholegrains, nuts, seeds, legumes, and seafood, and lower intakes of red or processed meats and fried foods^82^, ^83^, ^84^. The specific foods advised however will vary depending on the context and must be culturally and locally relevant.^82^, ^83^, ^84^ ^.^ In Arab countries for example, guidance suggests pregnant women follow general healthy eating guidelines, but consume adequate amounts of foods that contain iron as well as foods that promote the absorption of iron^85^.

Obstetricians and gynecologists should consider giving dietary advice during their routine clinical practice and, where available, women with obesity may benefit from consultation with a dietitian or specifically trained midwife^37^, ^77^, ^86^, ^87^. Nutrition and diet in pregnancy are addressed in more detail in FIGO’s “Think Nutrition First” recommendations^38^. In this article, detailed nutritional requirements are discussed in line with culture‐specific considerations. The FIGO PNCD Committee also developed a short dietary assessment tool for pregnancy: the FIGO Nutrition Checklist (supporting information S3). This short questionnaire addresses intakes of key nutrient‐dense foods for pregnancy and may assist obstetricians, gynecologists, and other healthcare practitioners briefly assess and advise on general healthy eating for pregnancy^22^, ^88^. This tool can be locally adapted based on the specific dietary practices of the region and relevant dietary guidelines and needs. Nutrition‐only interventions have been found to be superior to exercise‐only interventions for managing gestational weight gain during pregnancy, in normal weight and in overweight/obese pregnancy^89^. Therefore, dietary intervention may have the greatest potential for helping women achieve the appropriate gestational weight gain in pregnancy and this can be supplemented with counselling on physical activity^89^.

**Table:** 

B.2.2 Moderate intensity and appropriate exercise should be encouraged during pregnancy. **Strong** 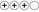

Women with obesity should be recommended to be physically active daily in line with local physical activity guidelines^37^, ^43^, ^61^. Although physical activity may help reduce excessive gestational weight gain, it is also associated with improved outcomes such as gestational diabetes and mode of delivery, independent of gestational weight gain^90^. The general global recommendation is to undertake a minimum of 150 minutes of moderate physical activity per week. Some member organizations recommend 30–60 minutes of moderate physical activity per day for women with obesity^86^. Consideration of the baseline physical activity levels of the woman should be made and, where required, advice given to build physical activity gradually to meet these recommendations in line with individual needs and capabilities^91^. The type of physical activity recommended will vary depending on the local context and the individual circumstances of the mother, however it could include types of moderate aerobic activity or strength conditioning exercise, aimed at maintaining fitness throughout pregnancy, rather than achieving peak fitness levels. The type of activity should also be selected so that there is minimal risk of falling from loss of balance or activity that could cause fetal trauma^64^.

**Table:** 

B.2.3. Women with obesity should continue to take folic acid during at least the first trimester. **Strong** 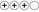

As is the case for all pregnant women, those with obesity should be encouraged to continue taking their folic acid supplementation daily throughout early pregnancy until the end of the first trimester^37^. A dose of at least 0.4 mg (400 μg) daily is recommended and consideration can be given to higher‐dose supplements as appropriate^37^, ^43^. Women with obesity are at higher risk of vitamin D deficiency due to deposition of the vitamin in adipose tissue deposits^92^. Therefore, while there is a lack of strong evidence to show that vitamin D supplementation can improve outcomes in women with obesity in pregnancy, to ensure that women with obesity are at least vitamin D replete, it is prudent to recommend a daily vitamin D supplement, in a safe dose, to women with obesity^37^, ^43^. In making the decision whether to supplement with vitamin D, the circumstances of the mother (e.g. diet or sun exposure) and the available resources should be considered. Additional nutrient‐specific or multinutrient supplementation may be indicated in certain regions or with women with limited dietary intakes. This may include iron, vitamin B12 or folate to prevent anemia, calcium to prevent pre‐eclampsia, iodine, or others as appropriate^44^, ^64^.

**Table:** 

B.2.4. Women with previous bariatric surgery require closer screening and monitoring of their nutritional status and fetal growth throughout pregnancy. They should be referred to a dietitian for advice about their nutritional needs and, where possible, have consultant‐led care. **Conditional** 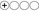

As discussed previously, women with a history of bariatric surgery are at risk of myriad vitamin and mineral deficiencies during pregnancy and are at higher risk of pregnancy complications^43^, ^56^. They should therefore have appropriate nutritional and other surveillance during pregnancy and receive consultant‐led care where possible^37^, ^43^. They should also receive sufficient nutritional care including supplementation appropriate for their specific needs and may benefit from dietetic support where available^37^, ^55^.

*Recommendation B.3. All women with obesity should be advised of the risk of obesity and excess gestational weight gain on pregnancy, childbirth, and long‐term health including risk of NCDs for them and their children.*

**Table:** 

B.3.1. All pregnant women with obesity in early pregnancy should be provided with accurate and accessible information about the risks associated with obesity and how they may be minimized. **Conditional** 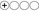

Recent evidence suggests that nearly a quarter (23.9%) of the risk of any pregnancy complication can be attributed to maternal overweight or obesity. In LMICs, the population‐attributable risk of overweight and obesity to gestational diabetes, gestational hypertension, and pre‐eclampsia is between 14%–35%^93^. Women with obesity should receive accurate and accessible information on the risks associated with obesity in pregnancy and the options for managing these risks. Risks to the mother of obesity during pregnancy include cardiometabolic disease (gestational diabetes, hypertensive disorders of pregnancy), sepsis, venous thromboembolism, stillbirth, preterm delivery, large‐for‐gestational‐age infant, and obstructive sleep apnea; for the fetus they include congenital anomalies or adverse birth outcomes including infant death^33^, ^44^, ^62^, ^93^, ^94^, ^95^, ^96^, ^97^, ^98^.

**Table:** 

B.3.2. Women should be informed that some screening processes for chromosomal anomalies are less effective in obesity. **Strong** 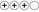

Obesity impacts scan completion and image quality, which can affect the detection of fetal anomalies^99^, ^100^. Women with obesity should be made aware that some forms of screening may less effective with a raised BMI.^37^, ^46^, ^101^, ^102^

**Table:** 

B.3.3. All pregnant women should be advised individually on mode of delivery, considering the risk of emergency cesarean delivery. **Conditional** 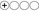

Obesity is not an indication for cesarean delivery; however, mothers with obesity may be at an increased risk of requiring an emergency cesarean delivery and repeat cesarean deliveries^98^. All pregnant women should receive individual advice on mode of delivery, considering all other contributing factors^43^.

*Recommendation B.4. All antenatal healthcare facilities should have well‐defined multidisciplinary pathways for the clinical management of pregnant women with obesity including the identification and treatment of pregnancy‐related complications.*

**Table:** 

B.4.1. Where possible, healthcare facilities should have clearly defined pathways for the management of pregnant women with obesity. The adequacy of resources and equipment available should be considered when making decisions around care, especially for women with a BMI ≥40. **Conditional** 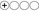

An environmental risk assessment, where feasible, should be completed and documented. Healthcare facilities providing maternity services should have a central list of the facilities and equipment required to care for pregnant women with obesity. These may include equipment such as larger chairs or wheelchairs. Individual practice locations should consider this in their planning and in line with their setting characteristics, storage space, and resource availability. Healthcare settings should consider audit and risk assessment as appropriate and possible within the setting^37^, ^46^, ^61^. Weighing women in their third trimester may support appropriate planning for resources requirements for labor and birth. This includes equipment that has specific weight restrictions, aspects of manual handling, and need for additional or specialist personnel, including theatre staff^37^, ^44^, ^46^, ^86^, ^103^, ^104^. Difficulties with venous access and anesthesia should be assessed and an anesthetic management plan for labor and birth should be discussed and documented. Healthcare professionals caring for the patient should consider multidisciplinary discussion in case significant potential difficulties are identified^37^, ^103^. Where possible, women with a BMI ≥35 should have at least one consultation with an obstetrician during pregnancy^86^.

**Table:** 

B.4.2. Women with obesity with multiple gestations require increased surveillance and may benefit from consultation with a maternal–fetal medicine consultant. **Strong** 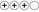

Multiple gestations increase risk of pregnancy complications and, therefore, further augment the risk associated with obesity in pregnancy, including the risk of pre‐eclampsia, gestational diabetes, and preterm birth^43^.

**Table:** 

B.4.3. All pregnant women with a BMI ≥30 should be screened for gestational diabetes in early pregnancy. **Strong** 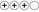

Early identification and treatment of pregnancy complications can help lessen their impact on immediate and longer‐term maternal and child health^27^. All pregnant women should be screened for gestational diabetes according to local and organizational practice guidelines^26^, ^105^. For more information on the identification, diagnosis, and management of diabetes in pregnancy and GDM, see the FIGO initiative on gestational diabetes mellitus: a pragmatic guide for diagnosis, management, and care^26^. In the case of diabetes in pregnancy, this may be identified any time during pregnancy and while GDM may also occur at any time, it is most likely after 24 weeks^26^. As a result, routine screening generally takes place in mid pregnancy, between 24 and 28 weeks of gestation^26^.

**Table:** 

B.4.4. Where available, an appropriately sized blood pressure cuff should be used for measurements. The cuff size used at the earliest time point should be documented in the medical records. **Conditional** 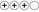

Cuff size influences the accuracy of blood pressure assessment, especially in those with overweight and obesity^106^. A cuff that is too small may overestimate blood pressure and lead to unnecessary medical intervention^107^. Where available, an appropriate size of cuff for the arm circumference of the woman should be used for all blood pressure measurements ^108^. Guidance exists from the American Health Association and other clinical practice guidelines recommend a large cuff for use when the mid/upper arm circumference is greater than 33 cm^27^, ^106^. Cuff size should therefore also be considered in conjunction with gestational weight gain and adapted as needed^109^. The cuff size used should be documented in the medical records at the earliest time point to allow for standardized assessment throughout gestation^37^, ^46^. Clinicians must take a pragmatic approach however and work with their available resources. This means it is reasonable to continue to follow their standard local practices where change of cuff size is not possible.

**Table:** 

B.4.5. To help prevent pre‐eclampsia, prophylactic aspirin from early pregnancy can be recommended to women with obesity that have other moderate to high risk factors. **Strong** 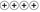

In recently published guidance, Poon et al.^27^ recommend all women receive screening for pre‐eclampsia in their first trimester using mean arterial pressure, in conjunction with maternal risk factors as a minimum. Obesity, especially a BMI of ≥35, is considered a moderate maternal risk factor for pre‐eclampsia^86^. Women with obesity and one or more additional moderate risk factors for pre‐eclampsia (first pregnancy, maternal age older than 40 years, family history of pre‐eclampsia, multiple pregnancy) may benefit from daily aspirin in early pregnancy (≤16 weeks) until birth, in line with local guidance^27^, ^110^. In higher‐resource settings, FIGO recommends that best practice for pre‐eclampsia screening include additional risk assessments such as uterine artery pulsatility index, placental growth factor, and serum pregnancy‐associated plasma protein A, although further evidence for the applicability of these biomarkers in all populations and ethnic groups, and to define their additional role in improving early prediction of preterm pre‐eclampsia is required at this stage. The relevance of these recommendations to LMICs is therefore uncertain and it is important that different countries modify the advice to be appropriate to the facilities and options available to them^27^.There is some evidence of a dose–response effect of aspirin in preventing pre‐eclampsia and a minimum dose of 100 mg has been suggested based on systematic review and meta‐analysis^111^. A dose of approximately 150 mg per day for women at high risk of pre‐eclampsia should be considered^27^.

**Table:** 

B.4.6. Clinicians should be aware that women with a BMI ≥30, before pregnancy or in early pregnancy, have a pre‐existing risk factor for developing venous thromboembolism during pregnancy. Risk of antenatal and postnatal venous thromboembolism should be assessed. **Strong** 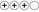

High maternal BMI is a risk factor for venous thromboembolism during pregnancy and postpartum^61^, ^112^. Prophylactic management should be considered for women with obesity in accordance with local and national guidance documents^103^, ^113^, ^114^, ^115^. This could include pharmacologic thromboprophylaxis, in appropriate doses based on maternal weight^37^, ^44^, ^46^, ^103^, ^104^.

**Table:** 

B.4.7. If available, women with a BMI ≥35 should be referred for serial assessment of fetal size using ultrasound as they are more likely to have inaccurate symphysis–fundal height measurements. **Conditional** 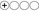

Serial measurement of symphysis–fundal height is recommended at each antenatal appointment from 24 weeks of gestation as this improves the prediction of a small‐for‐gestational‐age fetus^37^. It should be noted however that women with greater severity obesity (BMI ≥35) are more likely to have inaccurate symphysis–fundal height measurements^37^, ^43^. Where feasible, women with greater severity of obesity could be referred to ultrasound for serial measurement of fetal presentation^37^. This includes assessment of fetal weight and position at 35–38 weeks of gestation^86^.

**Table:** 

B.4.8. Due to the elevated risk of stillbirth associated with obesity, greater fetal surveillance is recommended in the third trimester in the case of reduced fetal movements. **Conditional** 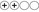

The risk of stillbirth in women presenting with reduced fetal movements increases with increasing maternal BMI. Increased surveillance should therefore be considered in women with obesity who have reduced fetal movements.^43^, ^116^

**Table:** 

B.4.9. Women with a BMI ≥30 are at increased risk of mental health problems, including anxiety and depression. Healthcare professionals should offer psychological support, screen for anxiety and depression, and refer for further support where appropriate and available. **Strong** 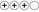

Mental health is a global priority for women^117^. Women with a BMI ≥30 are at increased risk of anxiety and depression^118^, ^119^. Some research suggests that there may be a dose–response relationship between maternal BMI and depression during pregnancy^120^. Women with obesity should therefore be screened for anxiety and depression in pregnancy^37^, ^44^, ^121^, ^122^. However, mental health issues should not necessarily be considered as contraindications to lifestyle interventions in pregnancy. Previous research in the UPBEAT randomized controlled trial found no association between antenatal depression status and adherence to healthy diet and physical activity advice^123^. If a woman is screened for anxiety or depression during pregnancy, further screening undertaken in the postpartum period should also be considered^124^. Intimate partner violence has also been associated with obesity.^125^, ^126^ Experiencing intimate partner violence is associated with weight gain over time and depressed mood further augments weight gained^127^. It is therefore prudent to suggest that women with obesity also be screened for intimate partner violence.

**Table:** 

B.4.10. Induction of labor is recommended at 41^+0^ weeks of gestation for pregnant women with a BMI ≥35 because of increased risk of intrauterine death. **Strong** 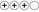

Elective induction of labor at term in women with obesity is associated with reduced odds of cesarean birth, macrosomia, and neonatal morbidity, without increasing the risk of adverse outcomes^37^, ^103^, ^128^, ^129^. Women with obesity may require longer labor induction with additional cervical ripening and oxytocin provision^130^. To reduce the risk of intrauterine death, induction of labor should be considered at 41^+0^ weeks of gestation for women with a BMI ≥35^86^. Decisions regarding induction or elective cesarean delivery should be made through individual discussion with the woman, taking her specific clinical and other considerations into account^37^. Induction of labor could also be considered in the case of suspected macrosomia as it associated with lower birth weight and fewer cases of shoulder dystocia or fractures^37^, ^131^.

**Table:** 

B.4.11. Women with a BMI ≥40 should be referred to an anesthetist for assessment in the antenatal period. **Conditional** 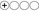

Anesthetic management of women with obesity may be more challenging and associated with higher risk due to increases in anesthetic time, issues with epidural insertion, and higher incidence of outcomes such as hypotension, heart rate decelerations, and airway intubation^103^, ^132^. Additional concerns based on the medical history of the mother may further complicate anesthetic management as is the case for obstructive sleep apnea, which increases the risk of adverse respiratory outcomes and sudden death^43^. Multidisciplinary discussion and planning should occur where significant potential difficulties are identified^37^, ^86^, ^103^.

Where possible, the on‐duty anesthetist should be informed of the admittance to the labor ward of a woman with a BMI ≥40. Early venous access should be considered during labor, especially for women with a BMI ≥40^37^, ^44^, ^104^. For women with a BMI ≥35 in active labor, electronic fetal monitoring is recommended^103^.

**Table:** 

B.4.12. Electronic fetal monitoring is recommended for women in active labor with a BMI ≥35. Intrauterine pressure catheters and fetal scalp electrodes may help. **Conditional** 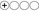

Women with more severe obesity (e.g. BMI> 35 for a European or American population) require extra vigilance on labor progress and close monitoring of the fetus^37^. Fetal scalp electrodes and ultrasound assessment of fetal heart can be used for fetal assessment^37^, ^103^. Intrauterine pressure catheters may help assessment of labor contractions^103^.

**Table:** 

B.4.13. In the case of vaginal delivery for women with a BMI ≥40, early placement of an epidural catheter is advisable in the case of an emergency cesarean delivery. **Conditional** 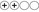

The insertion of an epidural is more challenging in patients with obesity. Early insertion may be advisable, depending on the clinical situation, and reduce anesthetic time^37^, ^103^.

**Table:** 

B.4.14. Establish venous access in early labor for women with a BMI ≥40 and consider a second cannula. **Conditional** 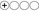

Women with obesity may receive a greater number of interventions during labor and birth compared to women with lower BMIs and may require additional time to achieve venous access^133^. For this reason, establishing early venous access may be beneficial^125^.

**Table:** 

B.4.15. Women with a BMI ≥30 having a cesarean delivery are at increased risk of wound infection and should receive prophylactic antibiotics at the time of surgery. Women with obesity may benefit from higher doses. **Strong** 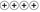

Obesity is a risk factor for pressure sores^134^. Women with obesity, in particular women with a BMI ≥40 in early pregnancy, should also have their tissue viability assessed using a validated scale in their third trimester and this documented to aid delivery management planning for prevention of pressure sores, including skin care and repositioning^37^. Constant care to prevent pressure sores and monitor fetal condition should be provided to women in established labor with a BMI ≥40^33^. Obesity is also associated with delayed wound healing^135^. Higher maternal BMI is associated with increased odds of wound complications, infections, and hemorrhage following cesarean delivery^136^, ^137^. Women with BMI ≥30 having a cesarean delivery should receive prophylactic antibiotics at the time of surgery^138^. Higher dose antibiotics may be beneficial^43^. To reduce the risk of wound complications, women with more than 2 cm subcutaneous fat should have suturing of the subcutaneous tissue following cesarean delivery^37^, ^86^, ^103^, ^139^. Subcutaneous drains can increase risk of wound complications and are therefore not recommended for routine use^44^.

**Table:** 

B.4.16. Active management of the third stage should be recommended to reduce the risk of postpartum hemorrhage. **Strong** 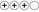

Active management of the third stage is recommended by the International Confederation of Midwives, FIGO, and WHO, among others, to reduce maternal blood loss^37^, ^140^, ^141^.

**Table:** 

B.4.17. Postoperative pharmacologic thromboprophylaxis should be prescribed based on maternal weight. **Conditional** 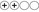

Venous thromboembolism is a major cause of maternal morbidity and mortality, particularly in the postpartum period^103^, ^142^. As outlined, prophylactic management should be conducted in line with local and national guidance documents and doses of pharmacologic thromboprophylaxis should be based on maternal weight, taking into account weight gain throughout pregnancy, and especially for women with a BMI ≥40^37^, ^44^, ^46^, ^103^, ^104^, ^113^, ^114^, ^115^. Postoperatively, continuing prophylactic treatment until the woman is mobile is suggested, however the optimal length of treatment is unknown and one should follow local guidance^103^, ^143^.

**Table:** 

B.4.18. Mechanical thromboprophylaxis is recommended before and after cesarean delivery. Women with a BMI ≥35 should be given graduated compression stockings, or other interventions such as sequential compression devices, after cesarean delivery until mobilization, which should be encouraged early. **Conditional** 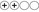

Women with a BMI ≥35 should be given graduated compression stockings, or other interventions such as sequential compression devices, in addition to low molecular weight heparin after cesarean delivery and until mobilization, which should be encouraged early^37^, ^86^.

## 5 FIGO Guidance for Postpartum Obesity

*Recommendation C.1. All women with prepregnancy obesity should receive specialist support on breastfeeding initiation and maintenance*.

**Table:** 

C.1.1. Obesity is associated with low breastfeeding initiation and maintenance. Women with obesity in early pregnancy should receive specialist advice on the benefits of breastfeeding and appropriate antenatal and postnatal support for breastfeeding initiation and maintenance. **Conditional** 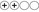

Women with obesity are less likely to initiate and maintain breastfeeding^144^. They should therefore receive appropriate advice and support antenatally and postnatally regarding the benefits of breastfeeding, considering the cultural and other needs of the woman^46^. Women can also be advised that breastfeeding, including exclusive and mixed breastfeeding, is inversely related to postpartum weight retention^145^. Women with obesity should also receive extra or specialist care antenatally to support breastfeeding initiation and maintenance if required^37^, ^44^. Psychological factors that are associated with breastfeeding behaviors in women include body image and social knowledge as well as beliefs on the nutritional adequacy and sufficiency of breast milk and the infant feeding preferences of others. Exploring these in women with obesity may support improved breastfeeding behaviors including improving body image and emphasizing the adequacy of maternal milk^146^.

*Recommendation C.2. All women with obesity and pregnancy complications should receive appropriate postnatal follow‐up in line with local resources, care pathways, and in response to the individual health requirements of the woman and her children.*

**Table:** 

C.2.1. Women who have been diagnosed with gestational diabetes and other pregnancy complications should have appropriate postnatal follow‐up. **Conditional** 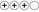

Higher risk of cardiovascular disease is seen in women who experience pregnancy complications such as gestational hypertension, pre‐eclampsia, gestational diabetes, placental abruption, preterm birth, and stillbirth and these independent risk factors therefore add to the cardiovascular risk associated with obesity^147^. This is especially important for LMICs that disproportionately share the burden of chronic diseases of cardiometabolic health. In low‐resource settings, cardiometabolic diseases may have earlier onset than in high‐resource settings. Women who develop pregnancy complications should have appropriate postnatal follow‐up to support the prevention, detection, and timely management of chronic diseases^37^. This should be completed 6–12 weeks after birth as soon as possible in the context of the resource setting and include aspects relevant to the complication experienced during pregnancy. For more details, see the FIGO Postpregnancy Initiative: Long‐term Maternal Implications of Pregnancy Complications—Follow‐up Considerations^21^.

**Table:** 

C.2.2. Due to the increased risk associated with obesity, where available, women with obesity should be screened for postpartum mental health disorders such as depression and anxiety. **Strong** 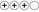

As discussed previously, healthcare professionals should be aware of the relationship between mental health and obesity and offer psychological support and referral where appropriate^44^, ^46^. Research has shown that prepregnancy obesity is associated with higher risk of postpartum mental health issues including depression^103^, ^148^. In addition, depressive symptoms in women with obesity have been shown to increase with increasing weight gain^149^. It is therefore prudent to recommend that, where available, women with obesity should be screened for anxiety and depression in the postpartum period as part of routine postpartum care and using a standardized tool, appropriate to the setting, where available^103^, ^124^.

*Recommendation C.3. All women with obesity should be encouraged to lose weight post partum with emphasis on healthy diet, breastfeeding if possible, and regular moderate physical activity. They should be advised of the importance of long‐term follow‐up as they and their children are at increased risk of NCDs.*

**Table:** 

C.3.1. Women should be informed that weight loss between pregnancies reduces the risk of stillbirth, hypertensive complications, and fetal macrosomia in subsequent pregnancies. Weight loss increases the chances of successful vaginal birth after cesarean delivery. **Strong** 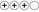

Postpartum health visits should be used as an opportunity to inform women about the benefits of weight loss between pregnancies, such as reduced risk of stillbirth, hypertensive complications, and fetal macrosomia, and increased chance of successful vaginal birth after cesarean delivery^37^. Weight loss in women with postpartum obesity, should be encouraged when indicated and appropriate, using advice and interventions that are suitable to the individual circumstances to the woman. Weight loss, achieved through diet and exercise interventions, is considered safe and can be encouraged for women who are breastfeeding^150^, ^151^. Lactating women can be advised that weight loss does not affect the quality or quantity of breast milk^152^.In women with postpartum obesity, weight loss should be encouraged regardless of gestational weight gained, as even modest weight retention after pregnancy is associated with increased risk of adverse outcomes^153^.

Postpartum weight retention of gestational weight gain is a significant contributor to weight status and risk of obesity at one year after birth in women, across all prepregnancy BMI categories^154^. Postpartum weight management, particularly in the first year after pregnancy, may therefore impact the long‐term weight status and weight gain trajectory of women, influencing their BMI in subsequent pregnancies^154^. Where postpartum weight loss is not possible or achieved, there should be an emphasis on the benefit of weight maintenance and the avoidance of additional weight gain after birth to reduce the risk of complications^44^. Intensive lifestyle interventions commenced in the postpartum period have been shown to support weight management^155^.

**Table:** 

C.3.2. Women with obesity should be offered further dietary advice to support postpartum weight management. **Conditional** 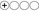

There is evidence that postpartum weight retention is mostly related to dietary intake rather than energy expenditure^156^. Interventions involving a combination of diet and physical activity therefore may be more effective than physical activity alone^46^, ^157^. Women with obesity should be offered personalized nutritional advice for weight reduction in the postpartum period from an appropriately trained professional as the first line in weight management^37^, ^44^. Refer to weight management services if available^37^.

*Recommendation C.4. Maternal obesity should be considered when making the decision regarding the most appropriate form of postnatal contraception.*

**Table:** 

C.4.1. Women with obesity should be counselled on the most appropriate form of postnatal contraception based on BMI. **Conditional** 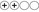

Due to the risks associated with pregnancy for women with obesity, preventing unwanted pregnancy is an important consideration^158^. Potential effects of various contraceptives have relevance to obesity and include increased risk of venous thromboembolism and weight gain^159^. Contraceptive counselling should consider these factors and any postpartum weight loss or retention that may affect fertility^46^. Efficacy of oral contraceptives in women with obesity does not seem to be affected, however the available evidence is of variable nature and quality^158^, ^160^, ^161^. Based on this, the risk of contraceptive failure with combined oral contraceptives cannot be excluded, particularly in those with a BMI ≥35^162^. Alternatives such as long‐acting reversible contraceptives, including intrauterine devices and subdermal hormonal implants or progestin‐only contraceptives, may be preferable where available and acceptable to the woman^159^.

## 6 Ethical Considerations for Obesity Management

The terms “obese” and “obesity” may be received negatively by patients due to the associated weight‐related stigma that is commonly experienced by people with obesity in society, including healthcare settings^44^, ^163^, ^164^. Stigmatization about weight can negatively influence mood, self‐esteem, and weight‐related behaviors, including food intake and physical activity. Experience of such stigmatization is associated with increased obesity, weight gain, and inflammation, ultimately influencing mortality and other outcomes^163^. Obstetricians and gynecologists should consider their individual bias toward women with obesity and take steps to address this so that they can offer the same respectful clinical care that women with lower BMIs receive^164^. It is unethical for clinicians in obstetrics and gynecology to decline to care for women who are otherwise within their scope of practice to manage, based solely on their BMI. If available however, referring to clinicians or clinical services with experience and capability in managing women with obesity may be appropriate^164^.

Many patients may identify with the concept of obesity as a disease process and as such, prefer “people‐first language”, as encouraged by the American College of Obstetricians and Gynecologists (ACOG) and used in other areas of health care including oncology^164^. Others, however, may disagree with this label and prefer the “health at every size” approach^165^. Obstetricians and gynecologists regularly see women with obesity owing to its high prevalence in many populations internationally. Therefore, clinicians caring for women with obesity before, during, and after pregnancy should consider the impact of their language on the individual. This document has been prepared in line with ACOG recommendations although obstetricians and gynecologists should consider the individual views and needs of the women they care for and adapt their language appropriately.

## 7 A Pragmatic Approach to Obesity Management in Women with Prepregnancy, Pregnancy, and Postpartum Obesity

The aim of this guidance on the management of prepregnancy, pregnancy, and postpartum obesity was to review the work of FIGO member organizations where it exists and consolidate evidence‐based clinical practice recommendations for widespread international application. As such, the current guidance is a review of published clinical practice guidelines. The PNCD Committee did not systematically review the evidence for each recommendation or details on qualifying data, therefore the individual clinical practice guidelines should be reviewed. The strength of this review, however, is that the perspectives and key considerations of different member organizations are outlined and synergies or differences discussed. This should be useful for other FIGO member organizations as they work to develop their own local or national guidance that considers the specific cultural and practical needs of their work setting. We encourage member organizations to use the guidance in this document as a framework to create and publish their own evidence‐based localized and pragmatic clinical practice guidelines, especially in LMICs that are currently under‐represented in the literature and have specific needs in relation to obesity that warrant special consideration. Throughout this article, BMI ≥30 is used as the definition of obesity. Although obesity assessment based on BMI is convenient and relevant, BMI alone is crude and other measures such as body composition or anthropometrics may be useful in understanding the impact on health^38^. Nonetheless, BMI is a simple‐to‐measure indicator that can be measured by a variety of trained staff and is therefore appropriate for use in a variety of clinical and other settings. A limitation of this review is that all of the clinical practice guidelines eligible to contribute to the key evidence‐based and graded clinical recommendations come from high‐income countries. This is because although other clinical practice guidelines exist, the evidence behind the outlined recommendations was not graded or the guidelines were not specific to this topic. Therefore, some of the advice throughout this article may not be applicable or possible to undertake in lower‐resource settings. Efforts were made to reference a broad range of international studies, where appropriate, and the corresponding international guidelines. We hope that by summarizing the available work of FIGO member organizations to date, in such a way, that LMICs can use this as a framework for the localization and adaptation of key clinical considerations for women with obesity before, during, and after pregnancy. There is also a clear need for further widespread research on the consequences and management strategies of obesity for women before, during, and after pregnancy in LMICs.

## 8 Summary

Managing obesity before, during, and after pregnancy may have widespread short‐ and long‐term benefits for mothers and their children. By addressing nutrition and weight with women of reproductive age, outcomes can be improved and the burden on healthcare systems reduced. The transgenerational effect of addressing weight with women is important to address the global burden of NCDs, which we argue are in fact communicable through the passage of risk from mother to child, across generations. This includes cardiometabolic diseases, respiratory diseases, and mental health conditions. Managing obesity will support the achievement of the UN Sustainable Development Goals and the promotion of population health, taking a life course approach to health promotion. As outlined, obesity management may include a variety of interventions from healthy diets, physical activity, and other medical or surgical options. Diet and lifestyle are the cornerstone of obesity management and while the degree of weight loss achieved with each intervention may vary, healthy diets and lifestyles should be encouraged and can further support additional interventions where employed. Even small amounts of weight loss may have positive effects on some pregnancy and other long‐term outcomes. During pregnancy, healthy diets and lifestyle can support management of gestational weight gain. Outside of weight management, women with obesity require specific considerations for medical, surgical, and other care planning and these are outlined in this review. While our objective was to formulate recommendations for the management of maternal obesity, the advice is a guide only and may not apply to all settings. The specific recommendations that are applicable to individual women or within resource settings may vary. Regardless of resource setting, we emphasize that clinicians consider obesity in all women of reproductive age before, during, and after pregnancy and advocate for healthy lifestyles for parents and offspring.

## Author Contributions

FMcA and SLK wrote the manuscript with contributions from all other authors. EH and SLK translated the evidence and recommendation grading for the review, based on the previously published clinical practice guidelines. All authors contributed to and reviewed the final manuscript.

## Disclaimer

Clinical judgement is paramount in decision making for the care of women with obesity before, during, and after pregnancy. Any recommendations outlined in this document should be considered in the context of the specific patient case. This advice must also be considered in line with relevant local, cultural, organization, and national guidance documents, requirements, policies, and practices. This guideline does not address all aspects of standard practice or individual patient care before, during, and after pregnancy. The responsibility for the comprehensive care of the patient lies with the healthcare professional, within their scope of practice. While the authors have endeavored to ensure that the content of this document is current at time of writing, it is up to the individual clinician to review future published research related to this document.

FIGO does not accept any liability to any persons for any loss or damage incurred because of reliance upon the advice included in this guideline summary and consolidation of recommendations from published national clinical practice guidelines of FIGO member organizations. For more details on the studies qualifying each of the recommendations, readers are directed to review the reference list and cited clinical practice guidelines.

## Conflicts of Interest

The authors declare no conflicts of interest.

## Supporting information


**Supporting information S1**. Overview of methods.

**Supporting information S2**. Evidence grading system.

**Supporting information S3**. FIGO nutrition checklist for pre‐pregnant/early pregnant women. Reproduced with permission from FIGO.
