# Supplementary material for: Management of prepregnancy, pregnancy, and postpartum obesity from the FIGO Pregnancy and Non‐Communicable Diseases Committee: A FIGO (International Federation of Gynecology and Obstetrics) guideline
Source: Int J Gynaecol Obstet. 2020 Sep 7;151(Suppl 1):16–36. doi: 10.1002/ijgo.13334 (PMC7590083; doi:10.1002/ijgo.13334)
Supplement: Supplementary file 1 — Supporting information S1. Overview of methods. [file IJGO-151-16-s001.docx]

**Supporting information**

**Supporting information S1:** Overview of methods.

The recommendations in this guideline were agreed by the FIGO Pregnancy and Non-Communicable Disease (PNCD) Committee in the following steps.

Approach and clinical priorities were agreed at a face-to-face Committee meeting in February 2019.

The Pregnancy Obesity and Nutrition Initiative (PONI) group undertook a review of published clinical practice guidelines specific to obesity in pregnancy through database searching and review of grey literature. Guidelines of most relevance to this document were agreed through consultation and all recommendations were extracted. Extracted recommendations were grouped by subject matter and consolidated through an iterative consultation process. The PONI group created 12 overarching recommendations to encompass all relevant extracted guidance from relevant international guidelines.

Overarching recommendations and associated details were agreed through wider PNCD Committee review at a face-to-face meeting in July 2019.

Further review of the literature was completed in February 2020 and the systematic review of Simon et al.^25^ was identified. Guidelines identified from this original search were cross-referenced against those found by this systematic review. New items were considered against the inclusion criteria.

The strength of the evidence for each extracted recommendation was noted in each of the individual clinical practice guidelines and appropriate terminology based on the GRADE criteria was applied, in line with other FIGO documents (see supporting information S2).

The manuscript was prepared based on agreed guidance and updated with recent evidence identified through database searching.

The final manuscript was agreed by all authors on July 23, 2020.
