# Supplementary material for: Management of prepregnancy, pregnancy, and postpartum obesity from the FIGO Pregnancy and Non‐Communicable Diseases Committee: A FIGO (International Federation of Gynecology and Obstetrics) guideline
Source: Int J Gynaecol Obstet. 2020 Sep 7;151(Suppl 1):16–36. doi: 10.1002/ijgo.13334 (PMC7590083; doi:10.1002/ijgo.13334)
Supplement: Supplementary file 2 — Supporting information S2. Evidence grading system. [file IJGO-151-16-s002.docx]

**Supporting information S2.** Evidence grading system.

| **FIGO levels of evidence** | **Levels of evidence in Clinical Practice Guidelines** | | | | |
| --- | --- | --- | --- | --- | --- |
|  | **SOGC ^43,103^** | **RCOG ^37^** | **DSOG ^86^** | **RANZCOG ^44^** | **ACOG ^46^** |
| **GRADE** | **Canadian Task Force on Preventative Health Care** | **N/A** | **Not provided** | **National Health and Medical Research Council (NHMRC)** | **Not provided** |
| High  | I – Evidence obtained from at least 1 properly randomized controlled trial | I ++ High-quality meta-analyses, systematic reviews of randomized controlled trials or randomized controlled trials with very low risk of bias  1+ Well-conducted meta-analyses, systematic reviews of randomized controlled trials or randomized controlled trials with a low risk of bias  1- meta-analyses, systematic reviews of randomized controlled trials or randomized controlled trials with a high risk of bias | A | A | N/A |
| Moderate  | II – 1 Evidence from well-designed controlled trials without randomization  II -2 Evidence from well-designed cohort (prospective or retrospective) or case-control studies, preferably from more than 1 center or research group | 2++ High-quality systematic reviews of case–control or cohort studies or high-quality case–control or cohort studies with a very low risk of confounding, bias or chance and a high probability that the relationship is causal  2+ Well-conducted case–control or cohort studies with a low risk of confounding bias or chance and a moderate probability that the relationship is causal | B | B | Level A  Good or consistent scientific evidence |
| Low  | II - 3 Evidence obtained from comparisons between times or places with or without intervention. Dramatic results in uncontrolled experiments (such as the results of treatment with penicillin in the 1940s) could also be included in this category | 2 – Case–control or cohort studies with a high risk of confounding, bias or chance and a significant risk that the relationship is not causal  3 Non-analytical studies, e.g. case reports, case series | C | C | Level B  Limited or inconsistent evidence |
| Very Low  | Opinions of respected authorities, based on clinical experience, descriptive studies or reports or expert committees | Expert opinion | D | D/Consensus | Level C  Consensus and expert opinion |

| **FIGO strength of recommendations** | **Levels of recommendations in Clinical Practice Guidelines** | | | | |
| --- | --- | --- | --- | --- | --- |
|  | **SOGC ^43,103^** | **RCOG ^37^** | **DSOG ^86^** | **RANZCOG ^44^** | **ACOG ^46^** |
| Strong | A | A,B | A,B | A,B | A |
| Conditional | B,C | B,C,D, Expert Opinion | D,C | C,D, Consensus | B,C |
